# Supplementary material for: IL-6 promotes tumor growth through immune evasion but is dispensable for cachexia
Source: EMBO Rep. 2024 Apr 26;25(6):2592–609. doi: 10.1038/s44319-024-00144-3 (PMC11169252; doi:10.1038/s44319-024-00144-3)
Supplement: Supplementary file 1 — Appendix [file 44319_2024_144_MOESM1_ESM.pdf]

IL-6 promotes tumor growth through immune evasion but is dispensable for cachexia

Young-Yon Kwon and Sheng Hui

Department of Molecular Metabolism, Harvard T.H. Chan School of Public Health, Boston, MA, USA

e-mail: shui@hsph.harvard.edu

Table of contents:

Appendix Figure S1. Characterization of cxC26 IL-6 KO subclone 2 (s2). **p2**

Appendix Figure S2. Validation of body weight loss and slow growth of the C26 IL-6 KO tumor in Balb/c mice. **p3**

Appendix Figure S3. Effects of *LIF* knockout in the cxC26 cells. **p4**

Appendix Figure S4. Inhibition of autocrine IL-6 signaling pathway by disrupting the IL-6 receptor in cxC26 cells. **p5**

Appendix Figure S5. Representative gating strategy for the evaluation of immune cell populations in the cxC26 scr and cxC26 IL-6 KO s1 tumors. **p6**

Appendix Figure S6. Expression of IL-6, LIF and IL-11 genes in the cxC26 scr and cxC26 IL-6 KOs1 tumors. **p7**

Appendix Table S1. List of guide RNA sequences designed for CRISPR/Cas9 constructs. **p8**

Appendix Table S2. Materials used for flow cytometry**.** **p8**

Appendix Figure S1. Characterization of cxC26 IL-6 KO subclone 2 (s2). CD2F1 mice were injected with saline, or inoculated with 1 X 10^6 cxC26 scr or 1 X 10^7 cxC26 IL-6 KO s2 cells. **A** Body weight. **B** Terminal muscle mass. **C** Tumor growth. **D** Circulating IL-6 concentration. All data are shown as the mean ± s.d. Significance of the differences: *P < 0.05, ** P < 0.01, *** P < 0.001 between groups by one-way ANOVA. ns, not significant. n = 5 for saline and cxC26 IL-6 KO s2, n = 6 for cxC26.

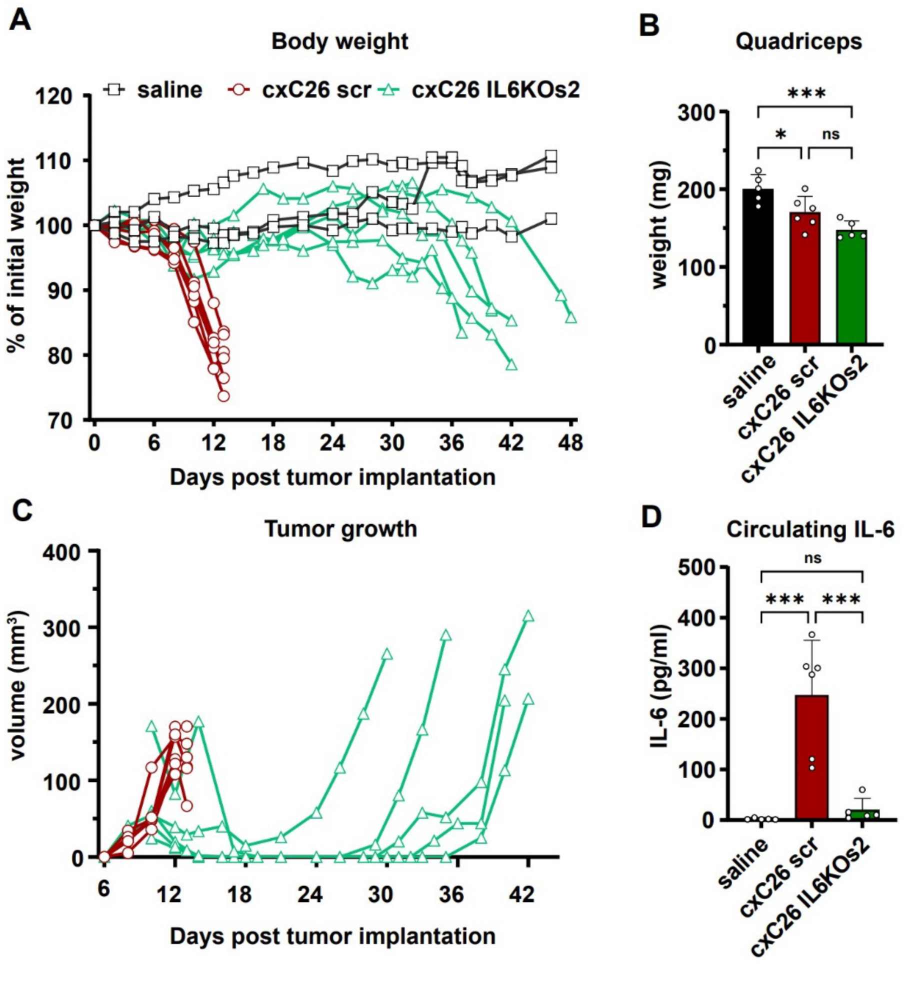

Appendix Figure S2. Validation of body weight loss and slow growth of the C26 IL-6 KO tumor in Balb/c mice. Balb/c mice were injected with saline, or inoculated with 1 X 10^6 cxC26 scr or 1 X 10^7 cxC26 IL-6 KO s1 cells. **A** Body weight. **B** Terminal muscle mass. **C** Tumor growth. **D** Circulating IL-6 concentration at the terminal time point. All data are shown as the mean ± s.d. Significance of the differences: *P < 0.05, ** P < 0.01, *** P < 0.001 between groups by one-way ANOVA. ns, not significant. n = 4 for saline, n = 8 for cxC26 scr, n = 4 for cxC26 IL-6 KO s1.

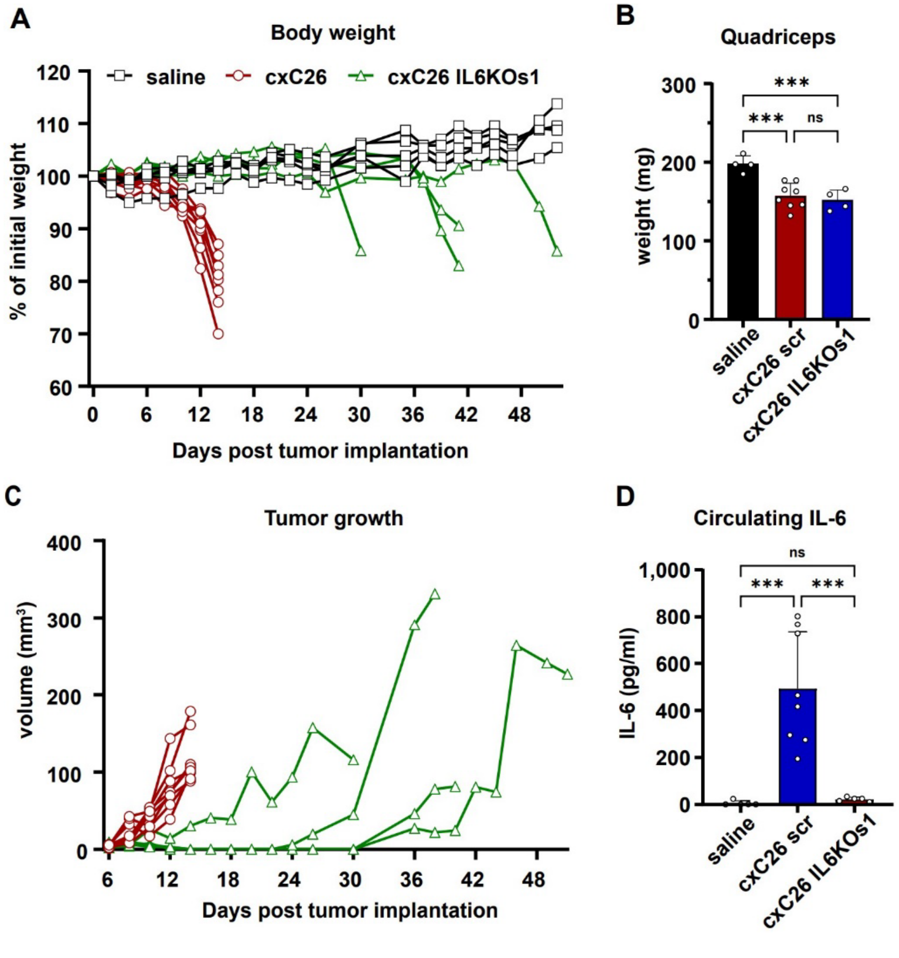

Appendix Figure S3. Effects of *LIF* knockout in the cxC26 cells. A Levels of LIF in conditioned media of cxC26 scr and cxC26 LIF KOp. **B** Body weight. **C** Tumor growth. **D** Terminal tumor mass. CD2F1 mice were injected with saline, or inoculated with 1 X 10^6 cxC26 scr or cxC26 LIF KOp cells. All data are shown as the mean ± s.d. Significance of the differences: *P < 0.05, ** P < 0.01, *** P < 0.001 between groups by Student's t-test. ns, not significant. n = 5 for saline, n = 7 for cxC26 scr, n = 7 for cxC26 LIF KOp.

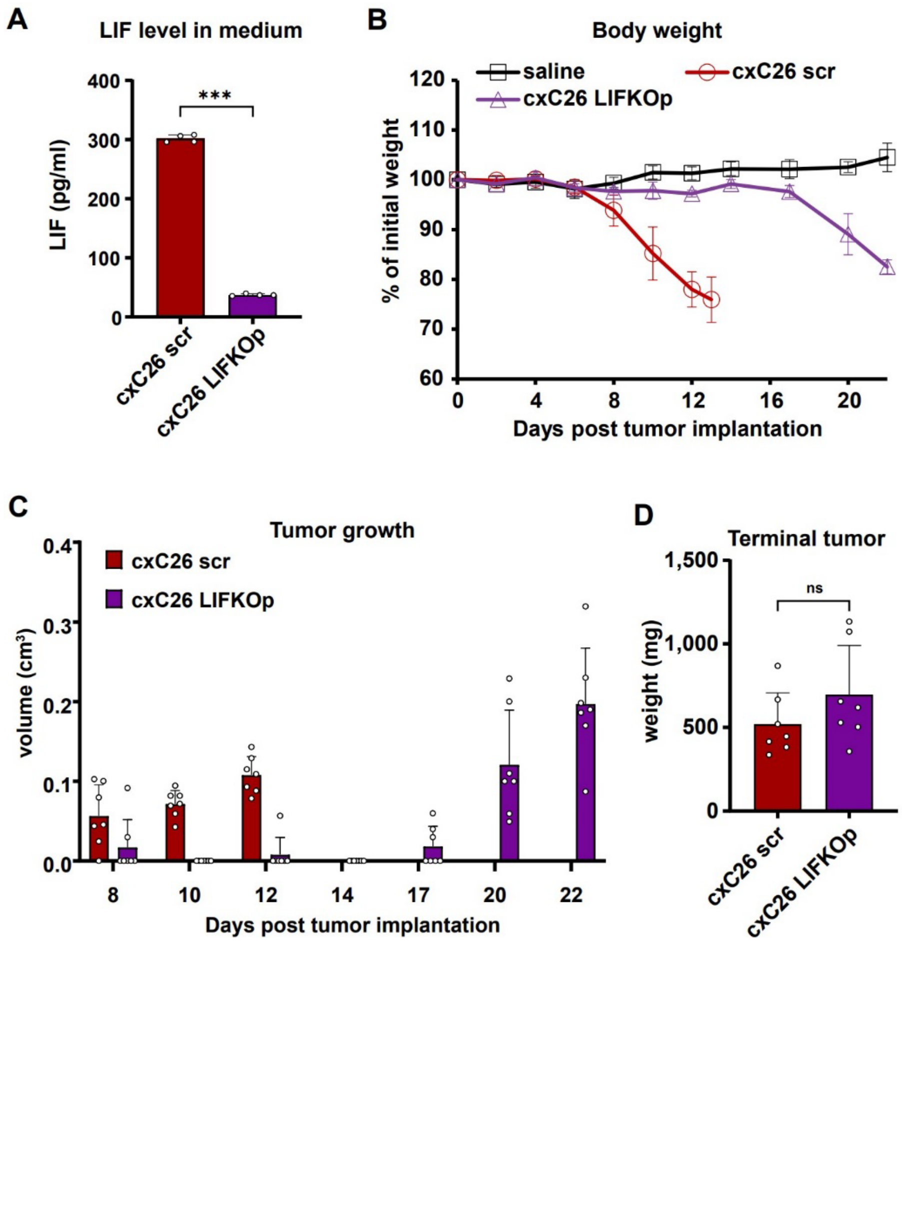

Appendix Figure S4. Inhibition of autocrine IL-6 signaling pathway by disrupting the IL-6 receptor in cxC26 cells. cxC26 IL-6 receptor α (*IL-6Rα*) knock-out pool (KOp) was constructed using CRISPR/Cas9. CD2F1 mice were injected with saline, or inoculated with 1 X 10^6 cxC26 or cxC26 IL-6Rα KOp cell. **A** Body weight. **B** Terminal tumor weight. Significance of the difference was between groups by Student's t-test. ns, not significant. n = 4 for saline, n = 5 for cxC26 scr, n = 6 for cxC26 IL-6Rα KOp.

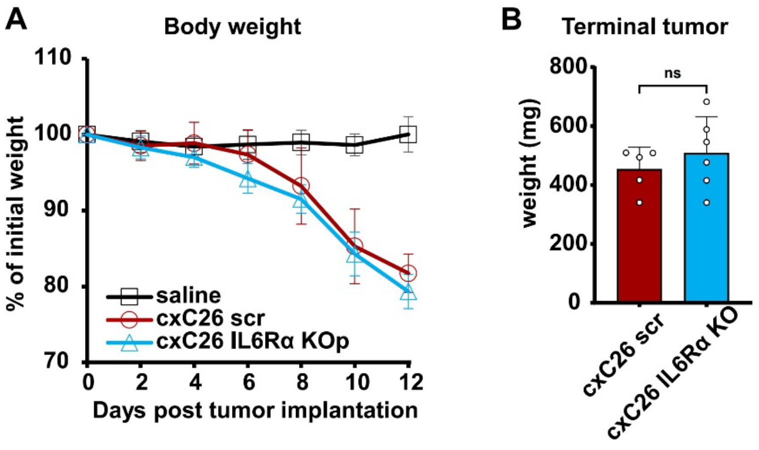

Appendix Figure S5. Representative gating strategy for the evaluation of immune cell populations in the cxC26 scr and cxC26 IL-6 KO s1 tumors. Tumors were harvested and dissociated into single-cell suspension and stained with various surface markers which are described in Appendix Table 2.

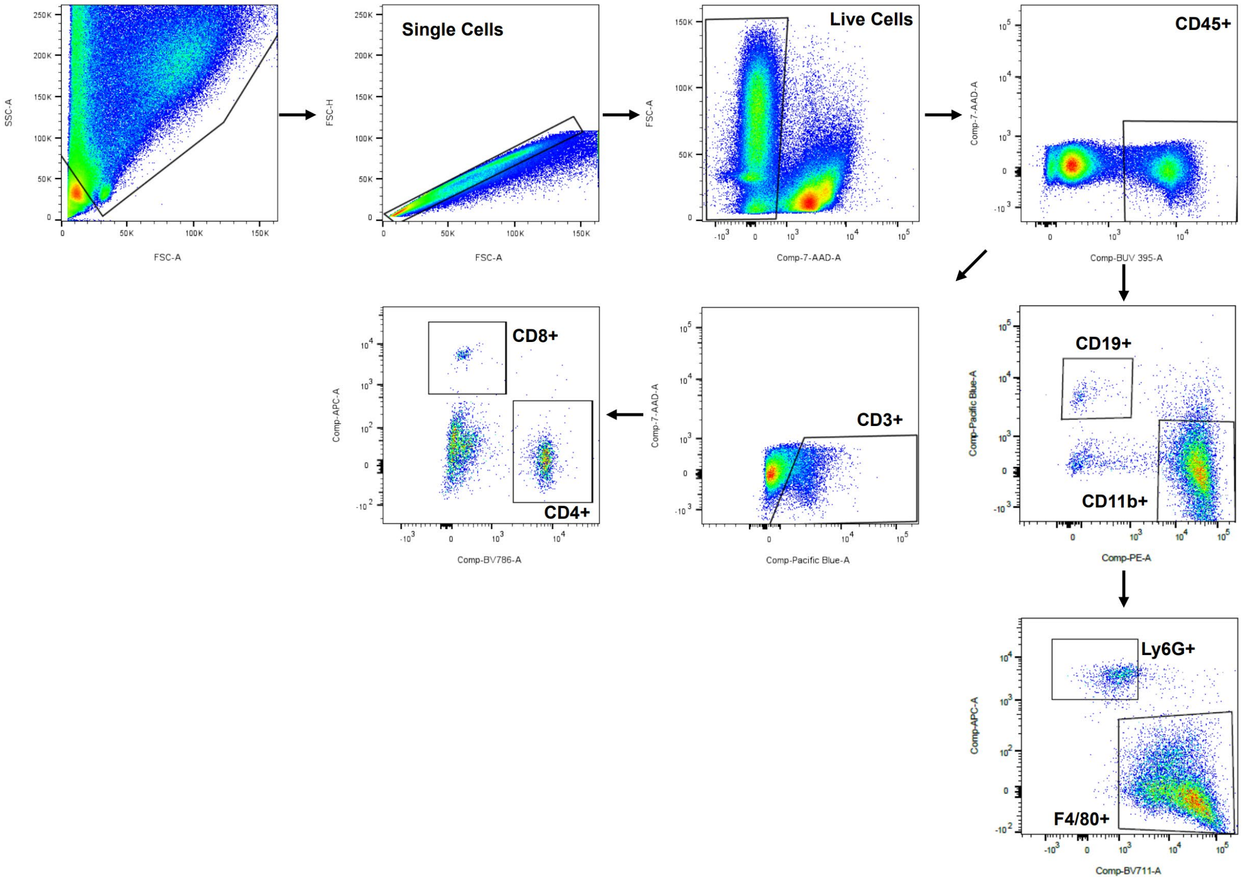

Appendix Figure S6. Expression of IL-6, LIF and IL-11 genes in the cxC26 scr and cxC26 IL-6 KOs1 tumors. Expression of IL-6, LIF and IL-11 genes in RNA-seq data were plotted following normalization of log_2CPM value using the limma-voom package. All data are shown as the mean ± s.d. Significance of the differences: *adjusted-P < 0.05, ** adjusted-P < 0.01, *** adjusted-P < 0.001 between groups by Student's t-test with FDR correction. ns, not significant. n = 4 for both groups.

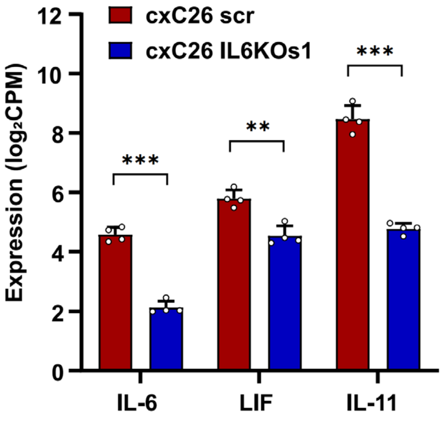

Appendix Tables

Appendix Table S1. List of guide RNA sequences designed for CRISPR/ Cas9 constructs.

Target		Sequences (5' -> 3')
IL-6	1	TCTGGAGTACCATAGCTACC
	2	TATACCACTTCACAAGTCGG
IL-11	1	GCGGAGTAGCCGTTCCAGTC
	2	CCATTGTACATGCCGGAGGT
LIF	1	TGCTACTATAGACGTCATGA
	2	CAAGTACCGTGTGGGCCACG
IL-6Rα	1	GGGGCAAATCAGGGTAACGG
	2	CTCACAGATGGCGTTGACAAG

Appendix Table S2. Materials used for flow cytometry.

Target	Clone	Fluorophore	Manufacturer	Cat#
**CD45**	30-F11	BUV395	BD bioscience	564279
**CD19**	1D3	Pacific blue	BioLegend	152415
**Ly6G+**	1A8	APC	BioLegend	127613
**F4/80**	BM8	BV711	BioLegend	123147
**CD11b**	M1/70	PE	BioLegend	101207
**CD3ε**	145-2C11	Pacific blue	BioLegend	100333
**CD4**	GK1.5	BV785	BioLegend	100453
**CD8a**	53-6.7	APC	BioLegend	100711
**Live/Dead**		7-AAD	BioLegend	420403
