# Supplementary material for: IL-6 promotes tumor growth through immune evasion but is dispensable for cachexia
Source: EMBO Rep. 2024 Apr 26;25(6):2592–609. doi: 10.1038/s44319-024-00144-3 (PMC11169252; doi:10.1038/s44319-024-00144-3)
Supplement: Supplementary file 10 — Expanded View Figures [file 44319_2024_144_MOESM10_ESM.pdf]

Expanded View Figures

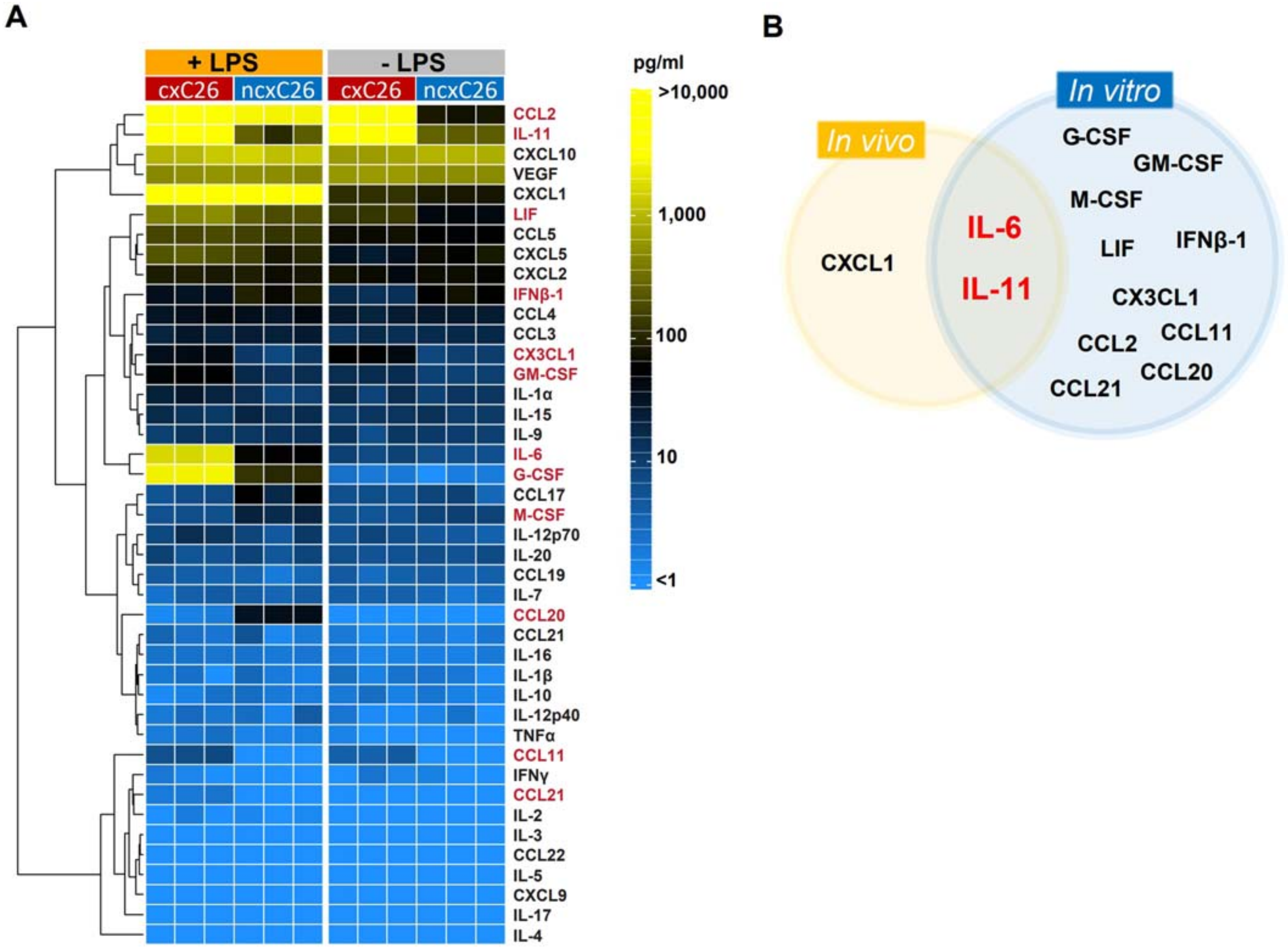

Figure EV1. Profiling of secretory cytokines from cxC26 and ncxC26 cells.

(**A**) Levels of cytokines in conditioned media with or without lipopolysaccharide (LPS, 2 μg/ml) treatment for 24 h. The measurement was done using the Luminex 44-cytokines panel. Cytokines highlighted in red are those with significantly altered levels under LPS treatment between cxC26 and ncxC26 (adjusted *p* value <0.01 by Student's *t*-test, $n = 3$ per group). (**B**) Comparison between significantly changed cytokines by cxC26 in in vivo (Fig. 1G) and in vitro.

© The Author(s)

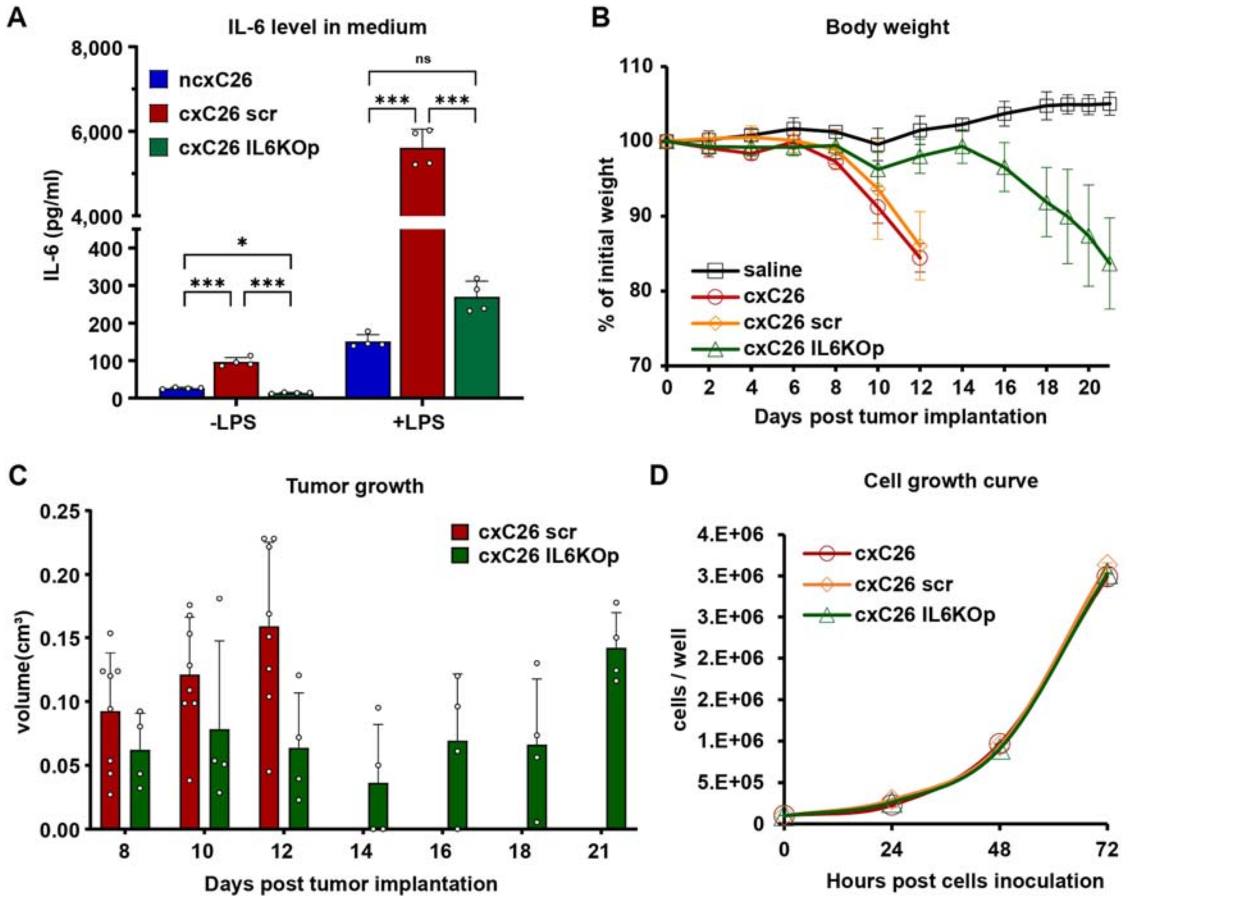

Figure EV2. Characterization of cxC26 IL-6 KO pool (IL6KOp).

(**A**) Levels of IL-6 in conditioned media of cxC26 scr (CRISPR/Cas9 scrambled gRNA control) and cxC26 IL-6 KOp with or without LPS (2 μg/ml) treatment for 24 h. (**B**) Body weight. (**C**) Tumor growth. CD2F1 mice were inoculated with 1×10^6 cxC26, cxC26 scr, or cxC26 IL-6 KOp cells. (**D**) Growth curves of cxC26, cxC26 scr, and cxC26 IL-6 KOp cells in in vitro. $n = 3$ per group. Data information: (**A**, **B**) $n = 4$ for per group. (**C**) $n = 8$ for cxC26 scr, $n = 4$ for cxC26 IL6KOp (**D**) $n = 3$ for per group. All data (**A**–**D**) are shown as the mean ± s.d. Significance of the differences: *$P < 0.05$, **$P < 0.01$, ***$P < 0.001$ between groups by one-way ANOVA. ns not significant.

 © The Author(s)

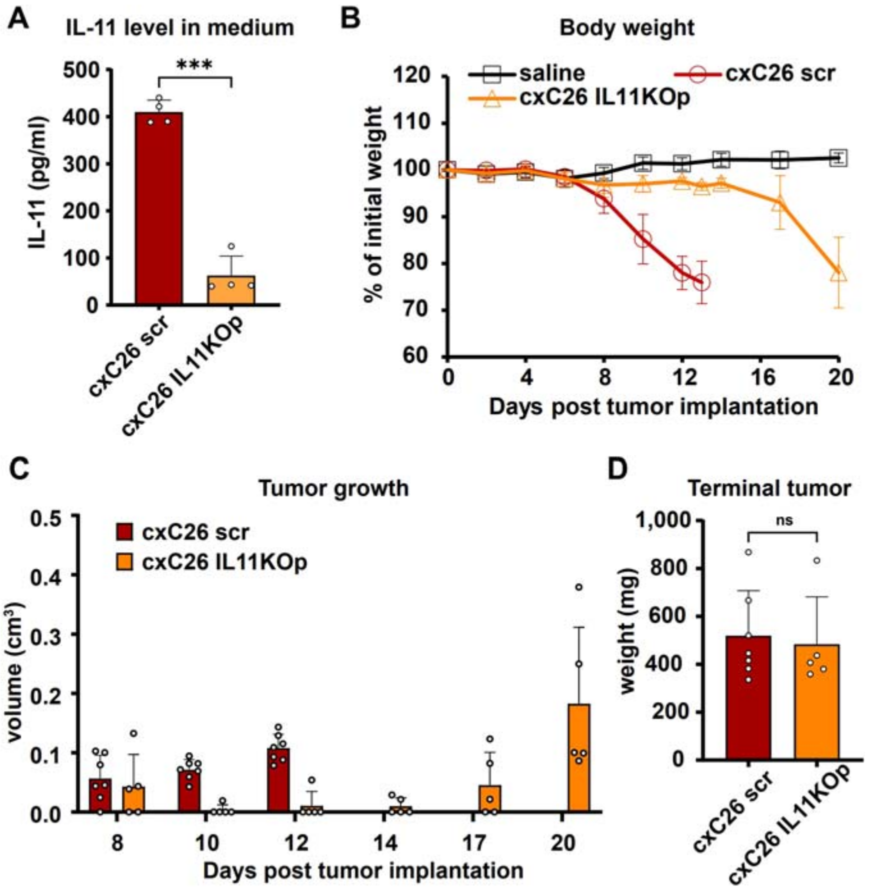

Figure EV3. Characterization of *IL-11* knockout in cxC26 cells.

(**A**) Levels of IL-11 in conditioned media of cxC26 scr and cxC26 IL-11 knockout pool (KOp). (**B**) Body weight. (**C**) Tumor growth. (**D**) Tumor mass at the terminal time point. CD2F1 mice were injected with saline or inoculated with 1×10^6 cxC26 scr or cxC26 IL-11 KOp cells. Data information: All data (**A–D**) are shown as the mean ± s.d. Significance of the differences: ***$P < 0.001$ between groups by Student's t-test. ns not significant. $n = 5$ for saline, $n = 7$ for cxC26 scr, $n = 5$ for cxC26 IL-11 KOp.

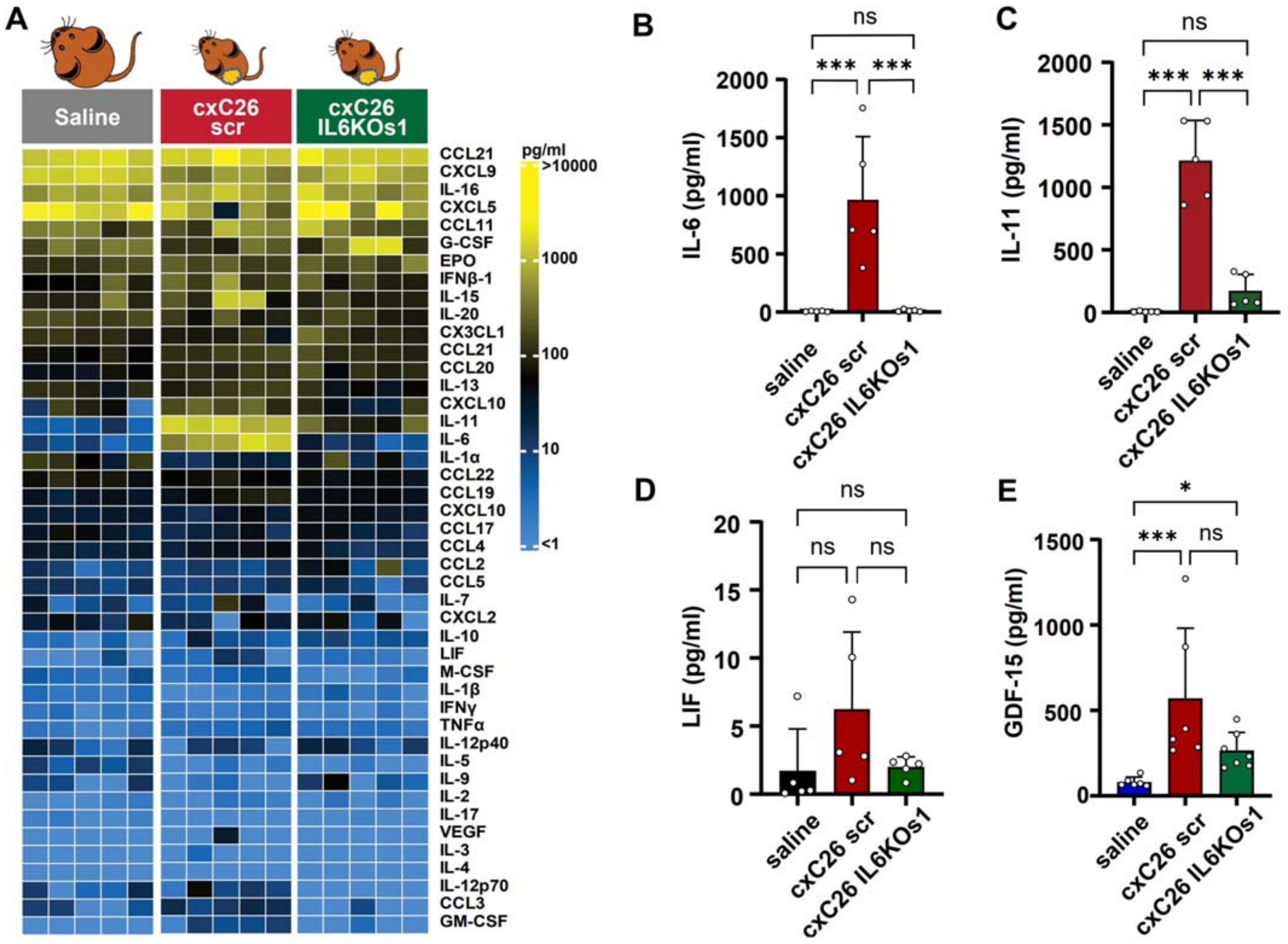

Figure EV4. Circulating cytokine profile in mice bearing the cxC26 IL-6 KO tumor.

CD2F1 mice were injected with saline or inoculated with 1×10^6 cxC26 scr or 1×10^7 cxC26 IL-6 KO s1 cells. (**A**) Heatmap for circulating cytokine levels and concentration of circulating (**B**) IL-6, (**C**) IL-11, and (**D**) LIF at the terminal time point. (**E**) Circulating GDF-15 concentration at the terminal time point. Data information: (**A–D**) $n = 5$ per group. (**E**) $n = 6$ for saline and cxC26 scr, $n = 7$ for cxC26 IL-6 KO s1. (**B–E**) are shown as the mean ± s.d. Significance of the differences: *$P < 0.05$, ***$P < 0.001$ between groups by one-way ANOVA. ns not significant.

© The Author(s)

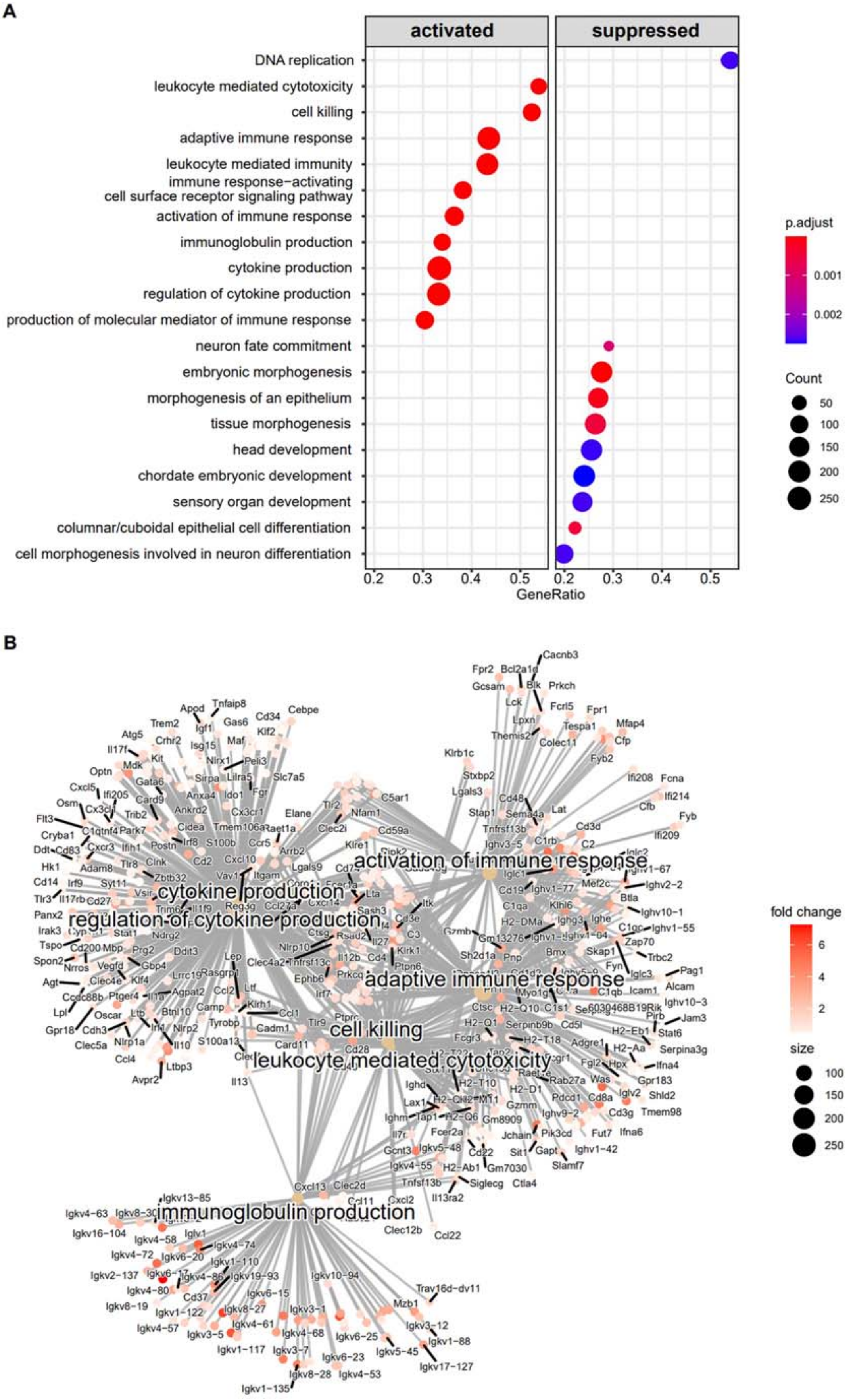
A
activated
suppressed
DNA replication
leukocyte mediated cytotoxicity
cell killing
adaptive immune response
leukocyte mediated immunity
immune response−activating cell surface receptor signaling pathway
activation of immune response
immunoglobulin production
cytokine production
regulation of cytokine production
production of molecular mediator of immune response
neuron fate commitment
embryonic morphogenesis
morphogenesis of an epithelium
tissue morphogenesis
head development
chordate embryonic development
sensory organ development
columnar/cuboidal epithelial cell differentiation
cell morphogenesis involved in neuron differentiation
0.2 0.3 0.4 0.5 0.2 0.3 0.4 0.5
GeneRatio
p.adjust
0.001
0.002
Count
50
100
150
200
250
B
cytokine production
regulation of cytokine production
activation of immune response
adaptive immune response
cell killing
leukocyte mediated cytotoxicity
immunoglobulin production
fold change
6
4
2
size
100
150
200
250

© The Author(s)

Figure EV5. Alteration of gene expression by disruption of IL-6 in the cxC26 tumor.

(**A**) Elevated and suppressed biological processes in the cxC26 IL-6 KO s1 tumor compared to the cxC26 scr tumor. (**B**) Network plot of the terms that are the most significantly changed biological processes in the cxC26 IL-6 KO s1 tumor. ClusterProfiler was used for calculating GeneRatio and adjusted *p* value (FDR) for identifying the significantly changed biological process and drawing the RNA-seq data. $n = 4$ for cxC26 scr and cxC26 IL-6 KO s1.

© The Author(s)
